# Novel Use of Proteomic Profiles in a Convex-Hull Ensemble Classifier to Predict Gynecological Cancer Patients’ Susceptibility to Gastrointestinal Mucositis as Side Effect of Radiation Therapy

**DOI:** 10.4172/jpb.1000363

**Published:** 2015-06-25

**Authors:** Ralph L Kodell, Randy S Haun, Eric R Siegel, Chuanlei Zhang, Angela B Trammel, Martin Hauer-Jensen, Alexander F Burnett

**Affiliations:** 1 Department of Biostatistics, University of Arkansas for Medical Sciences, Little Rock, AR 72205, USA; 2 Department of Pharmaceutical Sciences, University of Arkansas for Medical Sciences, Little Rock, AR 72205, USA; 3 Central Arkansas Veterans Healthcare System, Little Rock, AR 72205, USA; 4 Department of Applied Mathematics and Computer Science, Philander Smith College, Little Rock, AR 72202, USA; 5 Department of Obstetrics and Gynecology, Division of Gynecology Oncology, University of Arkansas for Medical Sciences, Little Rock, AR 72205, USA

**Keywords:** Cervical, Endometrial, Gastrointestinal mucositis, Prediction, Proteomics, Radiation

## Abstract

**Background:**

Whole-pelvis radiation therapy is common practice in the post-surgical treatment of cervical and endometrial cancer. Gastrointestinal mucositis is an adverse side effect of radiation therapy, and is a primary concern in patient management. We investigate whether proteomic information obtained from blood samples drawn from patients scheduled to receive radiation therapy for gynecological cancers could be used to predict which patients are most susceptible to radiation-induced gastrointestinal mucositis, in order to improve the individualization of radiation therapy.

**Methods:**

We use 132 proteins measured on 17 gynecological cancer patients in a convex-hull-based, selective-voting ensemble classifier to classify each patient into one of two classes: patients who would not (class 1) or would (class 2) develop gastrointestinal mucositis. We employ 20 repetitions of 10-fold cross-validation to measure classification accuracy.

**Results:**

We achieved a 95% confidence interval on average prediction accuracy of (0.711, 0.771) using pre-radiation proteomic profiles to predict which patients would experience gastrointestinal mucositis. Pathway analysis of the 12 most prominent proteins indicated that they could be assembled into a single interaction network with direct associations. The function associated with the highest number of these 12 proteins was cell-to-cell signaling and interaction.

**Conclusions:**

Pre-radiation proteomic profiles have the potential to classify cervical/endometrial cancer patients with high accuracy as to their susceptibility to gastrointestinal mucositis following radiation therapy. Further study of the network of 12 identified proteins is warranted with a larger patient sample to confirm that these proteins are predictive of gastrointestinal mucositis in this patient population.

## Introduction

In the current management of cervical cancer, all patients classified as stage IIb and above receive chemoradiation therapy. Roughly 50% of patients who are IIa and below also receive radiation. In most circumstances the radiation is given to the whole pelvis. For treatment of endometrial cancer, patients with positive pelvic lymph nodes are generally given pelvic radiation. Gastrointestinal mucositis (GM) is a common adverse side effect of radiation therapy, and is a primary concern in patient management. In this study, we investigate whether proteomic information obtained from blood samples drawn from patients scheduled to receive radiation therapy for gynecological cancers could be used to predict which patients might be most susceptible to radiation-induced GM. Successful prediction would suggest that patient management could potentially be improved by augmenting the use of conventional prognostic criteria with proteomic profiles for improved individualization of radiation therapy.

Advancements in biotechnology in recent years have increased the availability of high-dimensional data (e.g., genomic, proteomic, metabolomic) for biomedical decision making. For such data to be informative for patient care, it must be transformed from simply a mass of raw data on each patient to a higher level of relevant information. Statistical learning techniques have been used to develop computational algorithms that can process such high-dimensional data to classify unknown tissue or blood samples through supervised training on samples of known class [1]. The goal of these algorithms is to improve the assignment of therapies to patients in the treatment of disease, either by maximizing efficacy for the intended beneficial effect or by minimizing the risk of adverse side effects. In particular, we have recently developed a classification algorithm based on selective voting in convex-hull ensembles that improves classification accuracy in two-class problems having high-dimensional predictors [2].

Previously, we successfully used surface-enhanced laser desorption/ionization (SELDI) mass spectrometry (MS) to develop classification methods from patient serum with high sensitivity and specificity [3]. In this study we utilize liquid chromatography-tandem mass spectrometry (LC-MS/MS) to generate serum protein profiles for cervical/endometrial cancer patients scheduled to receive radiation therapy. We use our selective voting algorithm to predict the occurrence of GM following radiation treatment in these patients based on their pre-radiation proteomic profiles.

## Materials and Methods

### Clinical study

The clinical study was approved by the Institutional Review Board of the University of Arkansas for Medical Sciences (UAMS) under Protocol Number 113376. The study population consisted of subjects eighteen years of age and older who were treated for gynecological cancers in the Women’s Oncology Clinic at UAMS, and who were candidates for whole-pelvis radiation therapy following surgery. Recruitment occurred over a three-year period. There were no exclusions based on racial or ethnic backgrounds. Because of the minimal risk associated with the blood draw, subjects were not excluded based on medical criteria. Informed consent was obtained from all participants. Each participant had one 7.5 ml blood sample taken either at initial consultation in the clinic or at the pre-op consultation. Treatment to the whole pelvis included 4500 cGy delivered to standard fields via a 4-field technique with the exception of one patient who received a 2-field technique (AP, PA). Brachytherapy was employed in about two-thirds of the patients, and about one-half had chemoradiation therapy.

Study participants were required to complete three written questionnaires during the course of the study. These questionnaires asked for information on the frequency and severity of bowel movements, both before whole-pelvis radiation treatment began and subsequent to radiation treatment, to provide information for the diagnosis and grading of GM. The first questionnaire was completed when the subject consented to participate, the second approximately 4 to 8 weeks after radiation treatment began, and the third approximately 3 months after the second questionnaire. All three questionnaires were intended to be completed during regularly scheduled visits as part of the standard of care; however, in a few cases telephone follow-up was required for the third questionnaire. In all, twenty-three subjects were recruited to the study, of which seventeen completed all three GM questionnaires. GM was graded according to the NCI common terminology criteria for adverse events (CTCAE) [4]. All subjects had grade 0 (no GM) prior to radiation treatment. In this study, both Grades 1 and 2 were used as toxicity cutoffs for defining acute and chronic gastrointestinal mucositis. Grades 3 and 4 were not observed.

In addition to the proteomic profile derived from a subject’s blood sample, the following information was recorded for each participant: age, smoking status, body mass index (BMI), cancer diagnosis and stage, GM diagnosis and grade assessed upon completion of the third of three questionnaires by the participant, and medical history (prior surgery, other medical problems, medications).

### Proteomic data

At a regularly scheduled clinic visit (initial consultation or pre-op consultation), one vial of blood was obtained from each prospective participant either in the clinic or at a blood-draw station. A 7.5-ml sample was collected in a serum separator tube (SST) containing a polymer gel and clot activator for the preparation of a serum. The vial was transported to the proteomics laboratory where the blood in the SST tube was allowed to clot for 15 minutes and then centrifuged for 10 minutes at 1500 xg. The serum from the SST was then aliquoted into cryovials and immediately frozen at −70°C. Samples were thawed only once prior to processing for MS analysis.

To facilitate detection of lower abundance proteins, prior to LC-MS/MS analysis, a 10 μL aliquot of each serum sample was applied to a Pierce Top 12 Abundant Protein Depletion spin column (Thermo Scientific) to remove high-abundance proteins according to the manufacturer’s instructions. The spin column filtrates were lyophilized using a VirTis Advantage EL freeze dryer (SP Scientific), suspended in 100 μL of water, and desalted using Zeba spin columns (7K MWCO, Thermo Scientific) equilibrated with 100 mM ammonium bicarbonate. Solution digests were carried out in 100 mM ammonium bicarbonate (Sigma-Aldrich), following reduction in 10 mM Tris[2-carboxyethyl]phosphine (Pierce) and alkylation in 50 mM iodoacetamide (Sigma-Aldrich), by addition of 100 ng porcine trypsin (Promega) and incubation at 37°C for 12–16 hours. Peptide products were then acidified in 0.1% formic acid (Fluka). Tryptic peptides were separated by reverse phase Jupiter Proteo resin (Phenomenex) on a 100 × 0.075 mm column using a nanoAcquity UPLC system (Waters). Peptides were eluted using an 80 min gradient from 97:3 to 35:65 buffer A: B ratio. [Buffer A = 0.1% formic acid, 0.05% acetonitrile; buffer B = 0.1% formic acid, 75% acetonitrile.]. Eluted peptides were ionized by electrospray (1.8 kV) followed by MS/MS analysis using collision induced dissociation on an LTQ Orbitrap Velos mass spectrometer (Thermo). MS data were acquired using the FTMS analyzer in profile mode at a resolution of 60,000 over a range of 375 to 1500 m/z. MS/MS data were acquired for the top 15 peaks from each MS scan using the ion trap analyzer in centroid mode and normal mass range with normalized collision energy of 35.0. Proteins are identified from MS/MS spectra by database searching using the Mascot search engine (Matrix Science) with a peptide mass tolerance of 2 ppm, fragment mass tolerance of ± 0.5 Da, a maximum of 2 missed tryptic cleavages, and fixed carbamidomethylation of cysteine modification and variable deamidation and oxidation modifications. The Mascot results are uploaded into Scaffold 4 (Proteome Software) for viewing the proteins and peptide information. A 1% FDR is used as the cutoff value, and spectral counts are exported into an Excel spreadsheet. Two independent LC-MS/MS analyses were performed for each depleted serum sample. Ingenuity Pathway Analysis [5] was used to obtain disease associations of groups of proteins and their associated pathway interactions.

To develop predictors for the classification algorithm, the average of the two technical replicates was used to represent each protein in each sample. These averages were normalized according to the method of Byrum et al. [6] prior to modeling. In all, 235 proteins were identified in at least one of the 23 samples. For predictive modeling, it was decided to keep only the proteins for which at least 12 subjects had a non-zero average normalized value, which resulted in 132 proteins in the prediction set.

### Prediction algorithm

The selective-voting algorithm of Kodell et al. [2], with default settings, was used to predict, based on pre-radiation proteomic profiles, which subjects would experience radiation-induced GM and which would not. This algorithm is based on an ensemble of classifiers, where each member of the ensemble is defined by two class-specific convex hulls constructed from a pair of predictors measured on subjects in a training set. Here, subjects with grade-0 GM have been designated as class 1 (negative) and subjects with either acute or chronic grade-1 or grade-2 GM have been designated as class 2 (positive), and a pair of predictors is a pair of averages of normalized protein values.

To implement the algorithm, all possible pairs of proteins are initially considered, i.e., _132_C_2_ = 8646 pairs in this case. From these 8646 pairs, the *m* (*m* = 100 by default) best pairs in terms of highest values of R^2^ in a two-variable regression model fitted to the training samples are retained. For each retained pair of proteins, the *k* sample points in the training set may be represented by a two-dimensional plot in which separate convex hulls are formed for class-1 and class-2 samples. These convex hulls are trimmed to achieve complete separation. Then the ensemble member represented by a protein pair is considered to cast a vote to classify a sample, whether training sample or test sample, in class j (j=1 or 2) if that sample falls within the trimmed convex hull for class j. The ensemble member abstains from voting on samples that fall outside both trimmed convex hulls. Thus, a member of the ensemble votes to classify each subject into one of the two classes, or can abstain from voting on selective subjects. A simple majority vote by the members who do vote determines a subject’s classification. This algorithm performs as well as, or better than, a number of well-known classification procedures [2]. Its two-dimensional geometry exploits second-order interactions among predictors while being robust to the curse of dimensionality in that the training set needs to be populated with training-set points in only two dimensions at a time. Thus, datasets with small sample sizes but large dimensions can be handled without dimension-reducing transformations, so that identities of individual predictors (proteins) are naturally retained.

The cohort of seventeen subjects who completed all three GM questionnaires was used to assess the predictive ability of the proteomic profiles. The goal was to estimate the ability to predict the outcomes of samples not included in the dataset that was used to train the selective-voting ensemble. For this purpose, 10-fold cross-validation (CV) was employed. With 10-fold CV, a data set of *n* samples (here, *n* = 17) is randomly divided into 10 subsets each having (approximately) *n*/10 samples. For each subset, a classifier is trained on the remaining observations (the training set), and the trained classifier is then used to classify the samples in the subset (the test set). The combined value of the prediction accuracy or other performance index over the 10 test sets is the cross-validated estimate of that index. Twenty repetitions of 10-fold CV were performed based on different random permutations of the 17 samples in the data set. Because *n* = 17, in each repetition there were seven subsets of two samples each and three subsets of one sample each.

A number of different indices may be used to assess a classifier’s performance. For problems having a dichotomous outcome variable like the present study, the sensitivity (SEN: proportion of correct predictions among true positives), specificity (SPC: proportion of correct predictions among true negatives), positive predictive value (PPV: proportion of correct predictions among positive predictions) and negative predictive value (NPV: proportion of correct predictions among negative predictions) are indices that may be of interest in addition to the prediction accuracy (ACC: proportion of correct predictions among all samples) [7]. In the present application, these five performance measures were used. For each performance measure, the average (AVG), standard deviation (SD), and 95% margin of error (MOE) were estimated using the results of the twenty 10-fold CVs; calculations of MOE used asymptotic normality and the 97.5^th^ percentile of Student’s *t*-distribution with 19 degrees of freedom.

## Results

Table 1 contains the results of twenty 10-fold CVs for predicting the occurrence of GM in the seventeen subjects based on their profiles of 132 proteins. As can be seen, the average prediction accuracy (ACC) was 0.741 (74.1%), with considerable variability from CV-run to CV-run for the five performance measures (range in SDs: 0.061 – 0.080). There was little difference in average values among the performance measures, the range being 0.721 – 0.761. Over the twenty repetitions, one subject in one run (CV #10) was not classified because of a tied vote. Put another way, 339 of 340 samples (17 subjects × 20 repetitions) were classified by the selective-voting algorithm, with one abstention. Uncertainty in the performance-measure estimates of Table 1 can be quantified using 95% confidence limits, which can be calculated as AVG ± MOE using Table 1 values. For example, accuracy (ACC) in Table 1 has an AVG value (± MOE) of 0.741 ± 0.030, or (0.711, 0.771). Clearly, the confidence interval excludes 0.5 = 50% which corresponds to random chance prediction.

As a further check on the validity of our predictions, we randomly re-assigned GM class labels to the seventeen samples and ran another twenty cross-validations. As it turned out, eleven of the seventeen randomly assigned labels were correct (6 in class 1 and 5 in class 2) while six were incorrect (3 in class 1 and 3 in class 2). The average ACC over twenty CVs was only 0.394 (SD: 0.09). This poor accuracy is to be expected if the class labels are mixed up, and it gives assurance that our predictions are valid in the sense of reflecting truly predictive information found in the proteomic profiles [8].

An interesting feature of the data was that the occurrence of GM was statistically associated with the date enrolled on the study. While only one of the first eight subjects to be enrolled experienced GM grade 1 or 2, seven of the last nine subjects enrolled experienced GM grade 1 or 2 (Fisher’s exact test: p-value=0.0152). We could not identify any reason for this phenomenon. To our knowledge, nothing in our operating procedures changed during the course of the study. In our search for a possible explanation, we noted that there was a statistical association between BMI and the occurrence of GM. Eight of twelve subjects with BMI>30 had GM grade 1 or 2, while zero of five subjects with BMI<30 had GM grade 1 or 2 (Fisher’s exact test: p-value=0.0294). This is counter to expectations; that is, ordinarily subjects with higher BMI are expected to be less prone to experiencing GM from radiation treatment.

We could not establish a reason for the propensity for GM to be observed in subjects enrolled later in the study, other than the counter-intuitive positive association with BMI. To see what influence, if any, BMI might have on our protein-profile-based predictions, we added BMI as a predictor in the model, and we included age as well. The results of the predictions are given in Table 2, where three subjects were not classified across twenty CVs due to tied votes (one each in CV#s 6, 15, 17). The average ACC was little changed by adding age and BMI as predictors, and the variability range was comparable to using proteins only (SD range: 0.057 – 0.086), as was the variation among average performance measures (range: 0.732 – 0.768). If BMI is in fact positively associated with the occurrence of GM, it does not add appreciable predictive information above what is already provided by the protein profiles.

**Figure 1:**
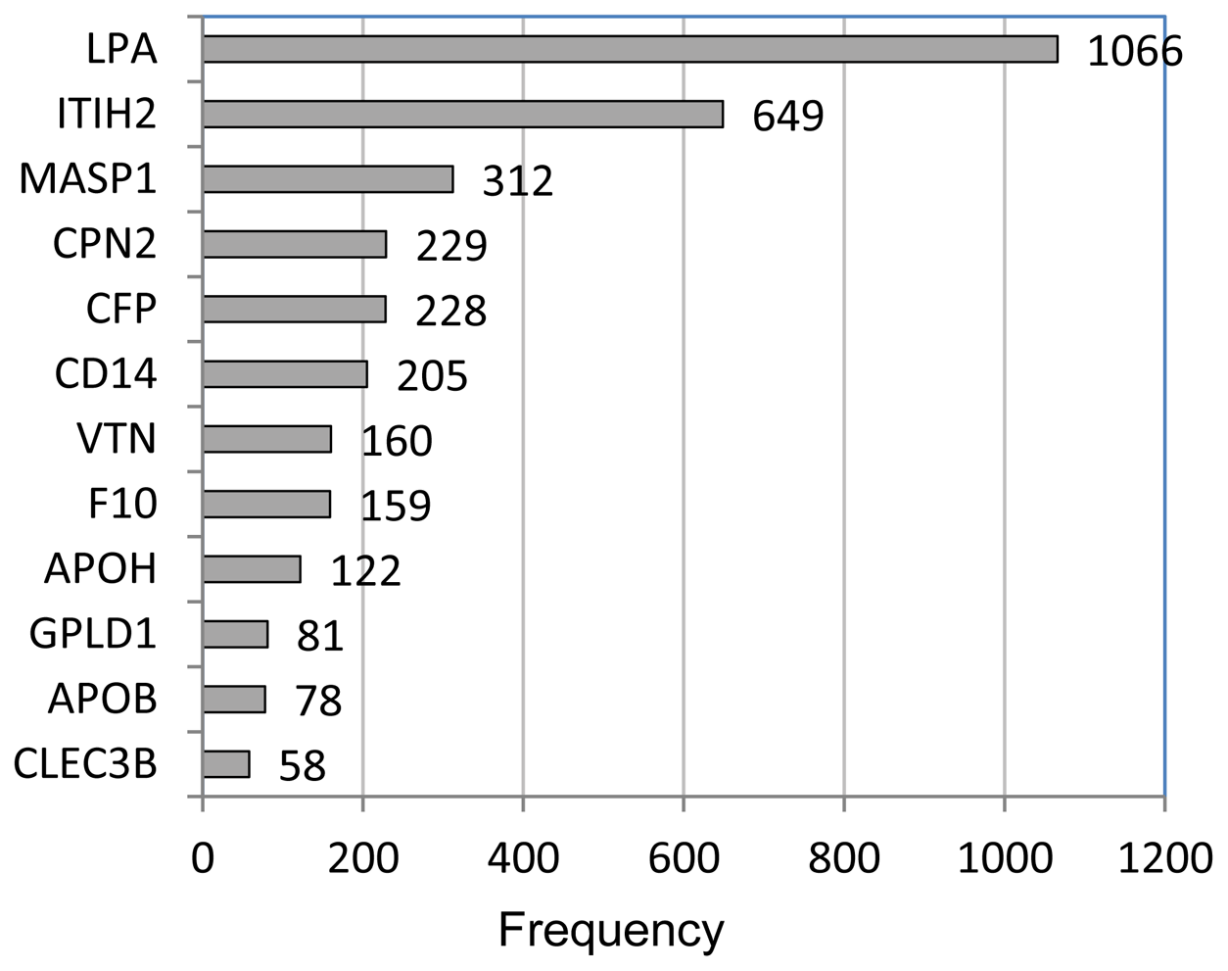
Frequencies (> 50) of occurrence of individual proteins in the set of 2000 best pairs from 20 runs of 10-fold cross-validation (100 per run). (Protein names in Table 3).

**Figure 2:**
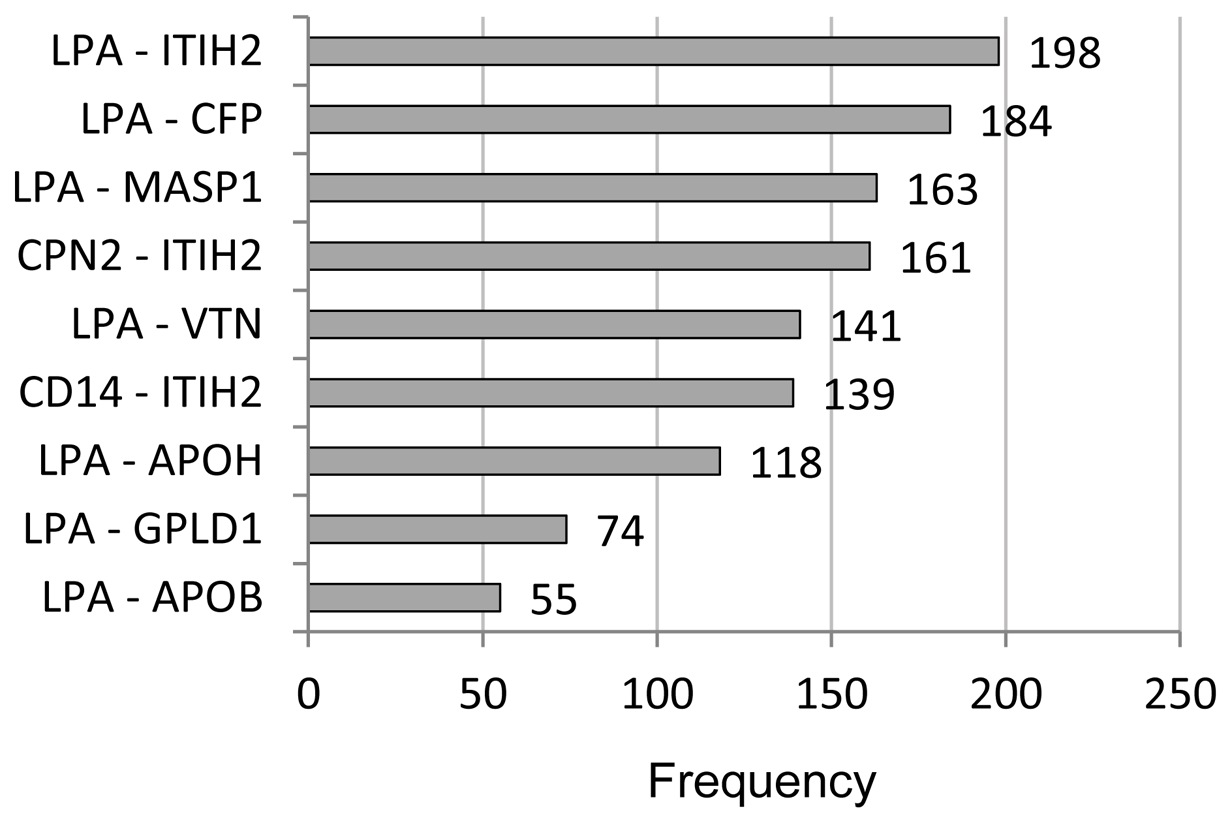
Frequencies (> 50) of occurrence of pairs of proteins in the set of 2000 best pairs from 20 runs of 10-fold cross-validation (100 per run). (Protein names in Table 3).

**Figure 3:**
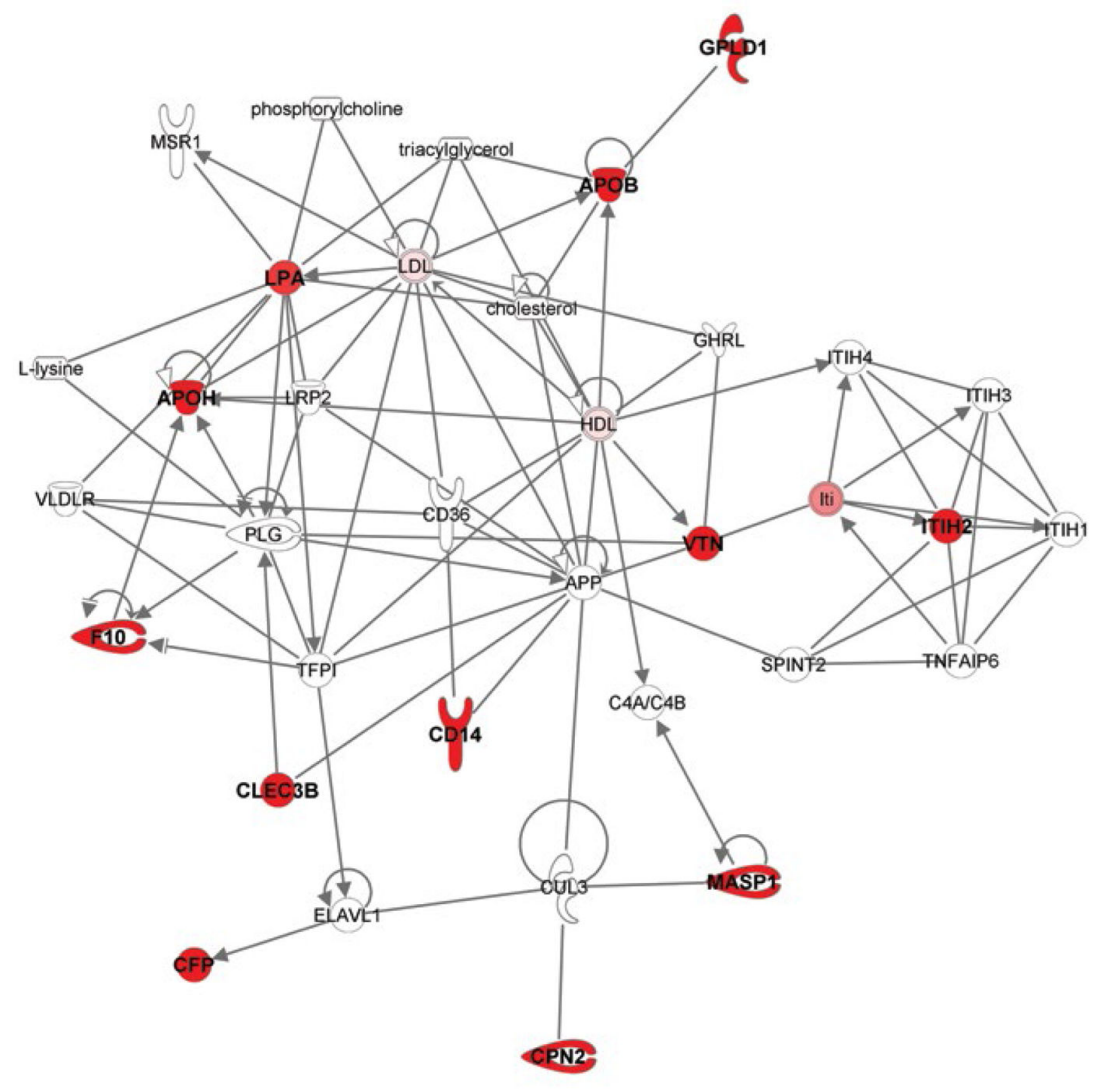
Network associated with the proteins in Table 3. The top associated network functions with these proteins are ‘hematological system development and function, cell-to-cell signaling and interaction, and organismal injury and abnormalities’ based on Ingenuity Pathway Analysis. Filled-in shapes represent proteins from Table 3; unfilled shapes represent relevant nodes/proteins added by Ingenuity. Solid lines connecting proteins indicate direct interactions.

From the CV results for the 132 proteins as predictors along with age and BMI (summarized in Table 2), the predictors occurring most prominently across the twenty runs were examined. Table 3 and Figure 1 identify the individual proteins and Figure 2 gives the protein pairs that were most prominent in the prediction of GM. In Table 3, the corresponding UniProt accession number and gene ID are given along with each protein name, as well as the mean and standard deviation of spectral counts for each of the two classes. In the figures and in our discussion, we refer to specific proteins via the gene ID. The ten protein-pairs with the largest R^2^ values in the regression phase of the selective-voting algorithm [2] in each block of each run of 10-fold CV (2000 in all) were first identified. Then the frequencies of occurrence of each individual protein and each protein-pair among those 2000 pairs were tabulated; proteins and protein-pairs with frequencies exceeding 50 were retained for Table 3 and the figures. Table 3 shows that for each of the top twelve proteins, spectral counts were higher on average in class 2 than in class 1, with some proteins showing high variability among subjects. As Figures 1 and 2 show, LPA (apolipoprotein (a)) and ITIH2 (inter-alpha-trypsin inhibitor heavy chain H2) were the two most prominent proteins, both individually and in pairs. Among the other ten prominent proteins, a high proportion of their individual frequencies can be explained by their pairing with either LPA or ITIH2, except for F10 (coagulation factor X) and CLEC3B (Tetranectin). LPA is involved in proteolysis, transport, lipoprotein metabolic processes, blood circulation, apolipoprotein binding, hydrolase activity, and endopeptidase inhibitor activity. ITIH2 is involved in negative regulation of peptidase activity, hyaluronan metabolic process, and endopeptidase inhibitor activity. Neither age nor BMI appeared among the most prominent predictors, in keeping with the lack of improvement in accuracy observed when they were added to the set of proteins (compare Tables 1 and 2). Pathway analysis applied to the 12 most frequently occurring proteins in the GM prediction model (Table 3) revealed an interaction network (Figure 3) with hematological system development and function, cell-to-cell signaling and interaction, and organismal injury and abnormalities as the top associated network functions.

## Discussion

Based on the clinical benefit of being able to determine which gynecological cancer patients might be susceptible to gastrointestinal mucositis following pelvic radiation, we sought to develop a proteomic-based classification algorithm for that purpose, and to identify potential proteomic biomarkers for at-risk patients. Our objective being to show proof of concept, rather than to compare performance of various classifiers, we have restricted attention to our convex-hull, selective-voting ensemble classifier to predict outcomes. Previously, we have shown our algorithm to be competitive with a number of well-known classification methods, including Random Forest, Boosting, k-Nearest Neighbor and Fisher’s Linear Discriminant Analysis [2]. To assess our classifier’s performance, we performed twenty repetitions of 10-fold cross-validation; a generally accepted method especially for high-dimensional predictor sets [9]. Our cross-validated results indicated prediction accuracy greater than 70% based on the 17 subjects and 132 proteins in our pilot study. Similar percentages were obtained for the other performance measures (Tables 1 and 2).

Table 4 divides the seventeen patients into GM-positive and GM-negative subgroups and provides a summary of clinical characteristics by subgroup. Four of the eight patients who developed mucositis had cervical cancer compared to seven of the nine who did not. The patient treated with the 2-field radiation technique did not develop mucositis. No clinical parameter typically associated with radiation-induced injury (smaller body habitus, cigarette smoking, concurrent chemoradiation), i.e., no conventional prognostic factor, was higher in the GM-positive group compared to the GM-negative group, and the percentages of patients receiving brachytherapy were virtually the same. Thus, the proteomic profile is more predictive of GM in this patient group than parameters typically associated with radiation-induced symptoms.

We note that Covington et al. [10] investigated the use of either an electronic nose with a K-nearest-neighbor classifier or field asymmetric ion mobility spectrometry with Fisher discriminant analysis to detect patients at risk of gastrointestinal toxicity during pelvic radiotherapy. Their prediction accuracies for both methods, as measured by leave-one-out cross-validation (*n*-fold CV where *n* is the sample size), were higher than ours. (Unlike 10-fold CV, *n*-fold CV cannot be repeated in order to assess a method’s variability.) However, their investigation included only patients who were at the two extremes of toxicity as measured by the IBDQ-B score. They selected 23 extreme cases from a larger cohort in order to achieve good separation of classes, and thus to be able to establish proof of principle. Acknowledging the built-in bias of this approach toward overstated accuracy, they mentioned that in future work they would include patients between the two extremes. In our study, none of the subjects experienced extreme toxicity, i.e., no subjects had grade-3 or grade-4 GM. Thus, our two classes were not markedly separated, and yet we were able to achieve average accuracy greater than 70%.

Interestingly, pathway analysis [5] of the 12 most prominent proteins in the prediction of GM indicated they could be assembled into a single interaction network with direct associations (Figure 3). The cellular function associated with the highest number of these 12 proteins was cell-to-cell signaling and interaction, related to the effects of five of these proteins (LPA, CD14, VTN, F10, and APOH) in binding of blood cells. Similarly, four of these proteins (LPA, VTN, F10, and APOH) are reported to be associated with hemostasis. Currently, it is unclear what role, if any, this network of proteins may play in predisposing an individual to GM or why they may serve as predictors of GM. However, further study is warranted with a larger patient sample to determine if these proteins are predictive of GM in this patient population. Importantly, to evaluate the specificity and sensitivity of the panel of biomarkers in such a study, a cohort of additional control subjects would be required to control for factors such as age, obesity, nutritional status, cholesterol levels, stress and medications.

Although limited because of sample size, the results indicate that selective voting in classification ensembles using proteomic profiles has the potential to classify cervical/endometrial cancer patients with high accuracy as to their susceptibility to the occurrence of GM following radiation therapy. However, we do not expect the MS-based method and the associated prediction algorithm to be used routinely for determining patient susceptibility if the biomarkers we have identified can be validated (and, perhaps augmented) in further studies. If we are successful in doing that, we envision that lab tests more relevant in clinical labs would be employed (e.g., ELISA). Once a panel of biomarkers is validated, the sophisticated data processing methods of this paper would not be necessary. For clinical practice, the positive predictive value (PPV) and negative predictive value (NPV) may be of most interest, as they indicate the likelihood that a patient is actually in the predicted class. Further development of this approach could provide the radiation oncologist with a valuable tool to help increase the precision of radiation levels for these patients.

A few subjects were left unclassified in the cross-validation runs due to tied votes among members of the ensemble. In practice, the prediction algorithm would not be applicable to such subjects, and they would need to be evaluated using different criteria. Fortunately, there were only three tied votes out of 340 attempts to classify when the algorithm used 132 proteins along with age and BMI as predictors, and only one tied vote when it used the 132 proteins alone.

We conclude that pre-radiation proteomic profiles have the potential to classify cervical and endometrial cancer patients with high accuracy as to their susceptibility to gastrointestinal mucositis following radiation therapy. Further study of the network of 12 most prominent predictive proteins is warranted with a larger patient sample to confirm their predictivity of gastrointestinal mucositis in this patient population.

## Supplementary Material

Supplementary File 1

Supplementary File 2

## Figures and Tables

**Table 1:** Prediction of occurrence of radiation-induced gastrointestinal mucositis based on pre-radiation protein profiles.^a^

CV	ACC^b^	SEN	SPC	PPV	NPV
1	0.706	0.75	0.667	0.667	0.75
2	0.706	0.75	0.667	0.667	0.75
3	0.706	0.75	0.667	0.667	0.75
4	0.706	0.75	0.667	0.667	0.75
5	0.824	0.875	0.778	0.778	0.875
6	0.765	0.75	0.778	0.75	0.778
7	0.647	0.625	0.667	0.625	0.667
8	0.706	0.75	0.667	0.667	0.75
9	0.706	0.75	0.667	0.667	0.75
10	0.812	0.75	0.75	0.857	0.778
11	0.765	0.75	0.778	0.75	0.778
12	0.765	0.75	0.778	0.75	0.778
13	0.765	0.75	0.778	0.75	0.778
14	0.765	0.75	0.778	0.75	0.778
15	0.765	0.75	0.778	0.75	0.778
16	0.882	0.875	0.889	0.875	0.889
17	0.765	0.75	0.778	0.75	0.778
18	0.765	0.75	0.778	0.75	0.778
19	0.706	0.625	0.778	0.714	0.7
20	0.588	0.5	0.667	0.571	0.6
AVG	**0.741**	**0.738**	**0.744**	**0.721**	**0.762**
SD	0.064	0.08	0.072	0.073	0.061
MOE	0.03	0.037	0.034	0.034	0.029

a Results of twenty 10-fold cross-validations. Class 1, GM grade 0: 9 subjects; class 2, GM grade 1 or 2: 8 subjects.

b ACC, SEN, SPC, PPV, NPV are defined in Methods. AVG, SD and MOE are the average, standard deviation and 95% margin of error of each performance measure over the 20 CVs.

**Table 2:** Prediction of occurrence of radiation-induced gastrointestinal mucositis based on pre-radiation protein profiles, age, and BMI.^a^

CV	ACC^b^	SEN	SPC	PPV	NPV
1	0.824	0.875	0.778	0.778	0.875
2	0.824	0.875	0.778	0.778	0.875
3	0.882	0.875	0.889	0.875	0.889
4	0.765	0.75	0.778	0.75	0.778
5	0.765	0.75	0.778	0.75	0.778
6	0.812	0.75	0.875	0.857	0.778
7	0.706	0.75	0.667	0.667	0.75
8	0.706	0.625	0.778	0.714	0.7
9	0.765	0.75	0.778	0.75	0.778
10	0.706	0.75	0.667	0.667	0.75
11	0.765	0.75	0.778	0.75	0.778
12	0.765	0.75	0.778	0.75	0.778
13	0.706	0.75	0.667	0.667	0.75
14	0.706	0.625	0.778	0.714	0.7
15	0.75	0.75	0.75	0.75	0.75
16	0.647	0.625	0.667	0.625	0.667
17	0.688	0.625	0.75	0.714	0.667
18	0.706	0.875	0.556	0.636	0.833
19	0.765	0.75	0.778	0.75	0.778
20	0.706	0.625	0.778	0.714	0.7
AVG	**0.748**	**0.744**	**0.752**	**0.732**	**0.768**
SD	0.057	0.086	0.076	0.064	0.064
MOE	0.027	0.04	0.036	0.03	0.03

a Results of twenty 10-fold cross-validations. Class 1, GM grade 0: 9 subjects; class 2, GM grade 1 or 2, 8 subjects.

b ACC, SEN, SPC, PPV, NPV are defined in Methods. AVG, SD and MOE are the average, standard deviation and 95% margin of error of each performance measure over the 20 CVs.

**Table 3:** Most frequently observed individual proteins.^a^

UniProt Accession No.	Gene ID	Protein Name	Class 1 Mean (SD)	Class 2 Mean (SD)
APOA	LPA	Apolipoprotein (a)	0.9 (1.2)^b^	5.8 (7.7)
APOB	APOB	Apolipoprotein B-100	26.2 (30.1)	79.4 (73.2)
APOH	APOH	Beta-2-glycoprotein 1	6.8 (5.2)	10.9 (6.7)
CD14	CD14	Monocyte differentiation antigen CD14	0.2 (0.5)	1.9 (1.7)
CPN2	CPN2	Carboxypeptidase N subunit 2	1.6 (1.9)	5.9 (6.0)
FA10	F10	Coagulation factor X	0.8 (1.0)	2,9 (3,5)
ITIH2	ITIH2	Inter-alpha-trypsin inhibitor heavy chain H2	14.7 (8.7)	32.4 (21,7)
MASP1	MASP1	Mannan-binding lectin serine protease 1	0.2 (0.4)	1.0 (0.9)
PHLD	GPLD1	Phosphatidylinositol-glycan-specific phospholipase D	1.3 (1.5)	3.5 (3.3)
PROP	CFP	Properdin	2.3 (1.1)	3.4 (1.6)
TETN	CLEC3B	Tetranectin	2.7 (1.8)	4.9 (1.1)
VTNC	VTN	Vitronectin	5.7 (2.9)	11.6 (6.2)

a Twelve proteins most frequently observed in the set of 2000 best pairs from 20 runs of 10-fold cross-validation (100 per run).

b Means and standard deviations (SD) of spectral counts.

**Table 4:** Clinical characteristics of positive and negative GM patients.

		Subgroup	
		GM positive (n=8)	GM negative (n=9)
BMI		45.0^a^ (30.6 – 66.3)^b^	34.1 (21.6 – 56.4)
Age		52.0 (26 – 76)	55.8 (30 – 75)
Past or current smoker		5^c^ (62.5)^d^	5 (55.5)
Chemoradiation		4 (50.0)	6 (66.7)
Brachytherapy		6 (75.0)	6 (66.7)
Cancer type	Cervical	4 (50.0)	7 (77.8)
	Endometrial	4 (50.0)	2 (22.2)

a Average,

b range,

c number,

d percent.
